# Food Co-Operatives: A Potential Community-Based Strategy to Improve Fruit and Vegetable Intake in Australia

**DOI:** 10.3390/ijerph17114154

**Published:** 2020-06-10

**Authors:** Seema Mihrshahi, Stephanie R. Partridge, Xiaolei Zheng, Divya Ramachandran, Debbie Chia, Sinead Boylan, Josephine Y. Chau

**Affiliations:** 1 Department of Health Systems and Populations, Macquarie University, NSW 2109, Australia; josephine.chau@mq.edu.au; 2 Prevention Research Collaboration, Charles Perkins Centre, Sydney School of Public Health, Faculty of Medicine and Health, The University of Sydney, Camperdown, NSW 2006, Australia; stephanie.partridge@sydney.edu.au (S.R.P.); shirleyz0827@outlook.com (X.Z.); divya.ramachandran@sydney.edu.au (D.R.); debbie.chia@health.nsw.gov.au (D.C.); sinead.boylan@sydney.edu.au (S.B.); 3 Westmead Applied Research Centre, Faculty of Medicine and Health, The University of Sydney, Westmead, NSW 2145, Australia

**Keywords:** fruit, vegetable, food co-operatives, diet

## Abstract

Only 5% of Australian children and adults eat enough fruit and vegetables. Two common barriers are high cost and limited access. Food co-operatives (‘co-ops’) may have the potential to reduce these barriers. We conducted a scoping analysis of food co-ops in the Sydney region to describe their characteristics and objectives. We also conducted a survey of members and non-members of co-ops to assess their fruit and vegetable intake using validated questions. Fifteen food co-ops were identified in the Sydney region and the most common objective was to provide cheap affordable produce. Most co-ops (61%) were in areas of high socio-economic status (SES). Members of food co-ops had a higher vegetable intake than non-members [mean difference (MD) = 0.54 serves/daily; 95% confidence interval (CI) of 0.15 to 0.93] and were also more likely to meet the recommendations for fruit and vegetable intake [odds ratio (OR) = 4.77 (95% CI = 1.15, 19.86)]. Implications of this study are that if food co-ops can be implemented on a wider scale, they hold potential for improving fruit and vegetable intakes.

## 1. Introduction

Diets low in fruit and vegetables are risk factors for cardiovascular disease, obesity, cancer and mortality [1]. The Australian Dietary Guidelines recommend that Australian adults consume at least two serves (150 g per standard serve) of fruit and at least five serves (75 g per standard serve) of vegetables each day [2,3]. In 2017–2018, only 7% of Australians aged 18 years old or over met recommendations for daily vegetable consumption, approximately half met recommendations for fruit consumption, and only 5% met the daily recommendations for consumption of both fruit and vegetables [4]. In 2017–2018, only 6.3% of children (age 2–17) met the daily recommended intake for vegetables, a larger proportion (73.0%) met the daily recommended intakes for fruit, and only 6.0% met the daily recommended intake for both fruit and vegetables [5]. The low level of vegetable intake, particularly in children, has remained constant over the past decade [6]. Effective strategies to increase fruit and vegetable intakes are urgently needed to address this problem.

Two commonly reported barriers to fruit and vegetable consumption are high cost and limited access [7]. People in areas of low socio-economic status (SES) are particularly at risk of having low intake of fruit and vegetables [8]. SES disparities in fruit and vegetable intake can be influenced by poor food environments, where higher availability of fast food and a lower availability of fresh food is apparent. Strategies to improve fruit and vegetable intake in families have mostly targeted the individual behavioural determinants of consumption and involved some form of nutrition education [9]. These approaches have had mixed success and evaluation periods are often short in duration and effect sizes can be small [9,10]. Other approaches include reduced-price incentives such as discounts or coupons for fresh fruit and vegetables which can significantly increase the sales and intakes [11].

Recognition of the wider range of influences on food preferences [12,13] means that a socio-ecological approach which creates supportive environments and better access to fruit and vegetables may be a more effective way of reaching populations with low intakes [14]. Community-based initiatives such as farmers markets [15], community gardens [16], cooking classes [17] and food co-operatives (‘co-ops’) [18] are examples of a socio-ecological approaches to improve healthy eating. Food co-ops are individual-, community- or charity-based groups, and enable their members to purchase fresh fruit and vegetables in bulk, resulting in cheaper and fresher produce directly from farmers or wholesalers [19,20]. Implementation of food co-ops may be an effective strategy for increasing fruit and vegetable daily intake among families living in Australia, especially in areas of low SES [19]. Evidence around the potential of food co-ops to improve fruit and vegetable intake is limited and most studies have been implemented in the USA. For example, a program called Brighter Bites [21] was conducted in low-income families and aimed to improve fruit and vegetable access and dietary education among school-based food co-ops [22]. The evaluation of Brighter Bites showed the program resulted in higher intakes as well as more positive attitudes in children for eating fruit and vegetables and demonstrated the feasibility and acceptability of the food co-op concept in this population [22]. Although food co-ops exist in Australia, information about them has not been systematically documented. This is important in order to determine whether this is a feasible and acceptable approach to improve intakes by removing the significant barriers of accessibility and affordability of fruit and vegetables in the Australian population.

Our research aims to explore the feasibility of establishing food co-ops in Australia with a view to improving fruit and vegetable intake. Firstly, we performed a scoping analysis to identify food co-ops in the Sydney metropolitan region with the aim of describing their characteristics, functions and objectives. Secondly, we undertook an online survey to examine whether adults (>18 years) participating in food co-ops in the Sydney metropolitan region differ in their fruit and vegetable intake compared to adults who do not participate in co-ops. We hypothesized that co-op members have a higher intake of fruit and vegetables than adults who do not participate in co-ops.

## 2. Materials and Methods 

### 2.1. Scoping Analysis

A scoping analysis (or review of the current situation) of food co-ops was conducted from October 2017 to June 2019 to identify those operating in the Sydney metropolitan area specified by the Australian Electoral Commission [23]. Formally defined, a co-op is ‘an autonomous association of persons united voluntarily to meet their common economic, social, and cultural needs and aspirations through a jointly owned and democratically-controlled enterprise’ [20]. Google and Facebook were searched (no specified dates) for content in English. Search terms were as follows: ‘food’, ‘fruit’, ‘veg *’, ‘Sydney’, ‘community’, ‘co-operative’, ‘cooperative’, ‘co-op’, and ‘school’.

Current food co-ops were identified, and their websites were explored. Co-ops were excluded if they did not sell fruits and vegetables, were located outside of the Sydney metropolitan area defined by the NSW Government [23,24], or information was not available in English. Data regarding the type, location, and objectives/purpose of each co-op, number of families participating and goods supplied were recorded. The suburb where each co-op was operating was recorded and we used the Socio-Economic Indexes for Areas—Index of Relative Socio-Economic Disadvantage (SEIFA-IRSD 2016) [24,25] to transform the postcode for the area into 10 deciles, with the 1st decile indicating the most disadvantaged and the 10th decile indicating the most advantaged area.

### 2.2. Online Survey

#### 2.2.1. Participants and Data Collection

We conducted an online survey using admin-approved advertisements in Facebook parent groups located in Sydney with at least 500 members. A convenience sample of adults who resided in the greater Sydney area, were aged >18 years old, and did not have any major health conditions were recruited to participate in an online survey using Research Electronic Data Capture (REDCap) tools hosted at the University of Sydney. REDCap is a secure web application for building and managing online surveys and databases. They were asked to read the Participant Information Statement online and written consent was obtained via a check box on the questionnaire before proceeding. The questionnaire collected demographic data (age, gender, indigenous status, marital status, number of children, highest level of education, employment status and language spoken at home) and used validated short questions about daily fruit and vegetable consumption (please see supplementary Table S1).

#### 2.2.2. Outcome Measures

Validated questions from the National Health Survey 2014–2015 [26] on fruit and vegetable intake were used to obtain a measure of serves of fruit and vegetables consumed per day. Participants were provided with an explanation about the definition of one standard serve based on the Australian Dietary Guidelines [2]. Participants were categorised into whether they met the Australian guidelines for fruit, vegetables and for both fruit and vegetables. Perception of current fruit/vegetable consumption was also measured (Table S2).

#### 2.2.3. Study Factor Measures

Participants were asked whether they were a member of a food co-op and if yes, they were asked a further four questions about the food co-op (Table S3). These included total number of members in the food co-op, types of food purchased from the food co-ops (fruit, vegetables and other food items), the frequency of buying a box from the food co-op and the amount (in Australian dollars) that participants usually spent per box. Because of the low numbers of food co-ops in the Sydney region generally, it was expected that there would be fewer members of a food co-op than non-members among the survey participants.

#### 2.2.4. Statistical Analysis

Demographic variables are presented as frequencies (*n*) and percentages (%) where categorical, and mean and standard deviation (SD) when continuous. To examine differences between groups, we used Pearson’s Chi-Square test for categorical variables and independent samples *t*-test for continuous variables. Three logistic regression models were created to examine the relationship between study factor and three binary outcomes (meet recommendations for fruit, vegetables and both fruit and vegetables combined). First, we conducted univariate logistic analysis for each explanatory variable; then, we fitted the baseline multiple logistic models including all covariates; next, we excluded each variable, except for the study factor, one by one from the highest *p*-value to the lowest *p*-value to determine the best fit of the model, after adjusting for context-specific variables (age and education), statistically significant variables and other potential confounders. Each model was presented separately based on the Hosmer–Lemeshow Goodness of Fit and Omnibus Tests of Model Coefficients. *p*-values of less than 0.05 were considered to be statistically significant. All statistical analysis was conducted by using IBM SPSS Statistics for Windows version 24.0 (IBM Corp, Armonk, NY, USA).

#### 2.2.5. Ethical Approval

Ethical approval for this study was given by the University of Sydney Human Ethics Committee (no. 2017/1016).

## 3. Results

### 3.1. Scoping Analysis

A total of 21 food co-ops were identified within the Sydney metropolitan area. One co-op (Bondi Food Collective) is no longer in operation, two co-ops were duplicates (The Radical Radish is part of the Harvest Hub; BOPCO is part of My Organic School), and three co-ops (Co-op Stop Network, Sydney Health Food Co-op, Broadway Food Co-op) did not include fruit and vegetables. These six co-ops were excluded, leaving 15 food co-ops for analysis (Table 1). Three of the co-ops operated across a number of sites within the Sydney metropolitan area. The Staples Bag focused on Western Sydney, with six locations providing fruit, vegetables and other food to the public. My Organic School is an initiative supporting parents from primary schools to start their own co-op and is, at the time of writing, operating in twelve schools. Harvest Hub (now called Sydney Farmers Collective) is described on its website as a “social enterprise food network” and has 61 locations in Sydney.

Seven co-ops (47%) supplied fruits and vegetables only and eight included other foods as well. Prices for boxes of food supplied ranged from $AUD12 for a small box to $AUD55 for a large box. Thirteen co-ops (87%) were not for profit and depended on volunteers. Five (33%) operated within schools and involved the school community. Only two co-ops relied on donated food from non-government organisations (NGOs) such as Foodbank [27]. A total of 20 of the 33 locations listed for the co-ops (61%) were in the higher SES areas (i.e., higher deciles 8–10) and only 5 (15%) were in areas of low SES (lower deciles 1–3).

There was a diverse range of objectives for setting up food co-ops. The most common objective was to supply affordable or cheap produce (80% of food co-ops). Ecological commitment (sustainable living, avoiding waste and packaging) was listed as a main purpose by 40% of the identified co-ops and another 40% mentioned quality/fresh produce and organic/pesticide-free or non-genetically modified food as a main purpose. Other objectives were to support local farmers (27%) and for good health (20%). Many of the school-based co-operatives included learning activities for children and they were involved in sorting the produce. The Staples Bag was specifically aimed at low-income populations. Rhubarb Food Co. was running educational workshops with a diverse range of topics (e.g., fermenting produce, lowering electricity bills, and relationships). Ignite Just Society was a church-based co-op and one of the main purposes was to support disadvantaged populations and activities included fundraising. The Inner City Family Food Co-op aimed to support families with access issues, in that many of the people that participated did not have a car. Green Gradz was set up to provide school children the opportunity and experience to run a business, and also as a social enterprise to support a school teacher for an orphanage in Thailand [28].

### 3.2. Online Survey Results

#### 3.2.1. Demographic Characteristics of the Study Population

A total of 402 eligible adults started the online survey, with 317 (79%) completing the survey and included in the analysis. Out of 317 study participants, 305 (96%) reported whether or not they have participated in a food co-op, and 12 (4%) participants did not provide an answer to this question. A total of 51 out of 305 (17%) of participants reported being part of a food co-op. Demographic variables are shown in Table 2.

There were some differences between the co-op members and non-members with respect to employment status. Members of food co-ops had a lower prevalence of being in full-time, part-time or in casual employment (75%) compared to the non-members (81%). Additionally, there were differences in the family size between groups, with the adults in food co-ops having more children (mean ± SD: 2.1 ± 0.83) than non-members (1.8 ± 0.90).

#### 3.2.2. Characteristics of the Food Co-Ops

Fifty-one (17%) of survey respondents were members of a food co-op compared to 254 (83%) who were not. There were 41 of 51 members who reported that they knew the number of members in the food co-op (10 missing observations) with an average of 14 people (SD: 8) in each food co-op. Almost all (98%) food co-op members purchased fruit and vegetables, while less than half (47%) bought other food items. The majority (69%) of the members of a food co-op collected a box once per fortnight spending an average AU$28 per box (SD: 10.7).

#### 3.2.3. Daily Serves of Fruit and Vegetables

More than half (55%) of the survey respondents consumed two or three serves of vegetables daily but only 8% met the vegetable recommendation (five or more serves of vegetables daily) (Table 3). The mean vegetable intake among the members of food co-ops (3.13 serves daily) was higher than non-members (2.58 serves daily) (t303 = 2.73; *p* = 0.007). The mean difference was 0.54 serves daily, with a 95% CI of 0.15 to 0.93 serves daily.

Most participants (62%) consumed one or two serves of fruit daily and just over half of participants met the fruit recommendation (two or more serves of fruit daily). The mean fruit intake among the members of food co-ops (1.71 serves daily) was slightly higher compared to that among people who did not participate in food co-ops (1.67 serves daily). However, this was not statistically significantly different (t303 = 0.253; *p* = 0.801). The mean difference was 0.041 serves daily, with a 95% CI of −0.275 to 0.356 serves daily.

#### 3.2.4. Perception of Fruit and Vegetable Consumption

More than a half (55%) of participants thought that they had ‘too little’ vegetables and 42% considered that they had enough serves of vegetables daily (Table 3). In contrast, almost 50% of participants thought that they had enough serves of fruit daily, with a slightly lower percentage (44%) of people considering that they should eat more fruit. A higher proportion (of members of food co-ops thought that they had enough serves of vegetables (49%) or fruit (59%) daily compared to non-members (41% for vegetables and 48% for fruit).

#### 3.2.5. Meeting Daily Recommendations for Fruit and Vegetables

Only 8% (*N* = 24) of participants met the Australian guidelines for five or more serves of vegetables daily but a much higher proportion (53% *N* = 160) of people met the Australian guidelines for two or more serve of fruits each day (Table 3). Combining both fruit and vegetable intake, we found that there were only 11 (4%) out of 305 participants who met the Australian guidelines for both fruit and vegetable consumption. Between the groups, a larger proportion of food co-op members met the guidelines for fruit, vegetable and both fruit and vegetable consumption (57%, 14%, 8%) than those who were not in a food co-op (52%, 7%, 3%).

Table 4 shows the logistic regression model examining the association between participation in food co-ops and meeting the recommendations for fruit and vegetable intake. Only 7% (17/254) of participants who were not in a food co-op met the recommended guideline, whereas 14% (7/51) of participants who participated in a food co-op met the recommended vegetable intake guideline. A total of 52% (131/254) of participants who were not in a food co-op met the fruit guideline, while 57% (29/51) of participants who participated in a food co-op met the recommended fruit guideline.

In terms of meeting both fruit and vegetable guidelines, there was a small percentage in both groups, with 4 (8%) out of 51 participants of food co-ops meeting the fruit and vegetable recommendation in daily intake compared to 7 (3%) in 254 participants who did not participate in food co-ops (*p* = 0.032). The adjusted odds ratio for whether members met the fruit and vegetable recommendation was 4.77 (95% CI = 1.15, 19.86), indicating members of food co-ops have more than 4-fold the odds of meeting the Australian guideline for both fruit and vegetables daily than non-members.

## 4. Discussion

Our study has provided a snapshot of the numbers and characteristics of current food co-ops operating in the Sydney metropolitan region. There were a diverse range of aims given for establishing food co-ops and the most common reason was to increase affordability. Interestingly, most food co-ops were located in high SES areas and there were less options in disadvantaged areas. Our study also demonstrated that members of co-ops had significantly higher intakes of fresh vegetables than non-members and there was also evidence that food co-op members were more likely to meet the Australian guidelines for combined fruit and vegetable intake than non-members. This finding may mean that establishing food co-ops on a wider scale may be an effective community-based strategy for improving consumption of fruit and vegetables.

Our study findings are similar to a study from Canada, which showed that fruit and vegetable consumption and overall diet quality was significantly improved for those who frequently shopped from food co-ops compared with people who purchased from other stores such as supermarkets [29,30]. In our study, food co-op members consumed half a serve of vegetables more than non-members. Although this seems a small increase, they also had more than 4-fold the odds of meeting the daily recommended intakes of both fruit and vegetables compared with non-members. On a population level, this would be significant and could lead to an increase in the current low level of compliance with the Australian guidelines. Higher proportions of members of food co-ops in our study also perceived that they ate the right amount of fruit and vegetables when compared to non-members, which may confirm that they were more likely to be complying with the guidelines for fruit and vegetable intake. In the Canadian study, members of food co-ops also tended to have higher perceived confidence that they consumed enough serves of fruit and vegetables compared with non-members [31] which is similar to the findings in our study. 

When considering how best to implement food coops on a wider scale, and in which regions, an important finding from our scoping analysis was that many co-ops operating in Sydney are based in areas of high SES. We recommend a targeted approach, establishing co-ops in areas of low SES, where people have a greater likelihood of having lower intakes of fruit and vegetables. It was also clear from our research that co-ops located in areas of disadvantage had a focus on affordability, a very important barrier to fruit and vegetable intake [7]. This would be especially important, for example, in the current COVID crisis where the prevalence of food insecurity is likely to increase. Recent research has shown that approaches implemented in low SES-communities that address the environmental as well as personal determinants of fruit and vegetable intake (such as mobile produce markets) are effective at improving fruit and vegetable intake and may have other benefits such as promoting social networks and engagement which is important for vulnerable populations [32]. Two-fifths of the co-ops in our study supplied only organic produce and 27% stated the importance of buying local from small Australian farmers to provide economic certainty and sustainability. This is consistent with previous research, where high-income earners are more likely to purchase commercial fruit and vegetable boxes for reasons of quality of produce, ecological commitment, purchasing local produce, and natural and organic production of food [33].

Another notable finding was that many of the food co-ops have been set up through schools; this may be an important leverage point to develop community-based activities around nutrition and healthy eating. In the USA, a school-based project ‘Brighter Bites’, which involved weekly distribution of fresh, donated produce from local food banks, resulted in significant improvements in fruit and vegetable intake over two months [21,22]. The study was organised through schools in low-income areas and also involved weekly recipe tastings, parental health education and distribution of resources such as handbooks and recipe cards. This approach was shown to be feasible and highly acceptable in low-income families and provided a variety of fruits and vegetables at a lower cost compared with most retailers [21]. Previous research suggests interventions targeted at improving diets are most effective in home and school settings [34].

One of the strengths of the cross-sectional survey is that we used validated questions from the Australian Health Survey [26] which have a high level of reliability and consistency and are widely used to measure fruit and vegetable intake in Australian adults. The study sample size was relatively large and representative of the general population, as evidenced by a similar prevalence of meeting the guidelines, even though participants enrolled online through Facebook. The study participant demographic characteristics are similar to those of the general population in the greater Sydney area. 

Limitations to the study include the cross-sectional survey design, which prevents drawing causal inferences between co-op membership and dietary intake, and this could be tested using an experimental study design. Other limitations include potential recall and measurement biases arising from self-reported consumption of dietary intake and potential confounders such as income were not measured. Our analysis was somewhat limited by the lower proportion of survey respondents who were part of a food co-op. However, this was expected because of the generally low number of co-ops operating in Sydney. In addition, convenience sampling from Facebook groups may limit generalizability, as some food co-ops may not have an online website or online presence.

## 5. Conclusions

Innovative solutions to increase fruit and vegetable consumption on a population level are urgently needed. Increasing fruit and vegetable intake can reduce the risk of cardiovascular disease, stroke and some cancers. Our research suggests that people participating in food co-ops tend to have a higher intake of fresh vegetables than non-members of food co-ops and are more likely to meet Australian guidelines for daily fruit and vegetable intake than non-members. This is important, as wide-scale implementation of fruit and vegetable co-ops could be an effective way of increasing fruit and vegetable intake in the community. We also found that food co-ops are acceptable, as there were diverse food co-ops already operating in the Sydney metropolitan region, with most in higher SES areas. Enabling communities to establish co-ops in areas of lower SES, for example by providing advice and resources, may facilitate implementation. Harnessing this community-based method may also result in wider benefits such as improving the food and social environment, creating new social networks, facilitating social engagement and enabling access to transport and fresh food markets.

## Figures and Tables

**Table 1:** Characteristics of the food co-ops identified in the Sydney Metropolitan Region.

Name (Website)	Location (Suburb)	Post Code	SEIFA-IRSD Decile *	FP/NFP †	Population Type	Products	Average Cost ($)	Source of Fruits and Vegetables	Form of Communication
USYD Food Co-op (usydfoodcoop.org)	Camperdown (University of Sydney)	2050	9	NFP	Uni students and staff, local community	F&V + Food	30	Markets	Online
Manly Food Co-op (manlyfoodcoop.org)	Manly	2095	10	NFP	Local community	F&V + Food	n/a	Farmers/Local Grower	In store
Alfalfa House (www.alfalfahouse.org)	Newtown	2042	9	NFP	Local community	F&V + Food	28	Farmers/Local Grower	In store and online
Thoughtful Foods (www.thoughtfulfoods.org.au)	Kensington (University of New South Wales)	2033	9	NFP	Uni students and staff, the local community	F&V + Food	35	Markets	In store
The Staples Bag (thestaplesbag.ssi.org.au)	Campsie	2194	2	NFP	Low-income earners in the local community	F&V + Food	32	Farmers/Local Grower	In store
	Surry Hills	2010	8						
	Campbelltown	2560	3						
	Redfern	2016	5						
	Parramatta	2150	6						
	Cranebrook	2749	6						
	Kingswood	2747	5						
	Blacktown	2148	4						
	Glebe	2037	7						
	Mt Druitt	2770	1						
	Baulkham Hills	2153	10						
Rhubarb Food Co-op (rhubarbfood.org.au)	Randwick	2031	10	NFP	Local community	F&V + Food	40	Markets	Online
Ignite Food Store & Op Shop (ignitefoodstore.com.au)	Mt Druitt	2770	1	NFP	Low-income earners in the local community	F&V + Food	25	Donated Food/Foodbank	In store
Harvest Hub (harvesthub.com.au)	Across Sydney (61 locations) North Turramurra	2074.	10	FP	Local community	F&V	44	Farmers/Local Grower	Online
Inner City Family Food Co-op	Alexandria	2015	10	NFP	Local community	F&V	25	Markets	Not known
Yeo Park Infants School	Ashfield	2131	6	NFP	School students’ families, staff	F&V	30	Markets	Not known
Madang Ave Public School Food Co-op	Mt Druitt	2770	1	NFP	School students’ families, staff	F&V	12	Markets	Not known
Spyns Inc. Food Co-op	Penrith	2750	5	NFP	School students’ families, staff	F&V + Food	15	Donated Food/Foodbank	Not known
Lilyfield Co-op (lilyfieldlife.com/2012/04/cheap-fresh-seasonal-fruit-and.html)	Balmain/Peninsula/Rozelle/Lilyfield (Balmain)	2041	10	NFP	Local community	F&V	25	Markets	Not known
Green Gradz	Bondi (Montessori East Primary School)	2026	10	NFP	School students’ families, staff	F&V	55	Farmers/Local Grower	Online, newsletter
My Organic School (myorganicschool.com/about)	Bronte, Waverley	2024	10	FP	School students’ families, staff	F&V	50	Organic farmers	Online
	Clovelly	2031	10						
	Rose Bay	2029	10						
	Paddington	2021	10						
	Woollahra	2025	10						
	Bondi Beach	2026	10						
	Balgowlah North	2093	10						
	Birchgrove Surry Hills (Bourke Street, Crown St Primary Schools)	2041	10						
		2010	8						

* Socio-Economic Indexes for Areas—Index of Relative Socio-Economic Disadvantage (SEIFA-IRSD 2016); decile 1 is most disadvantaged and decile 10 is the least disadvantaged (25); ^†^ NFP = not for profit; FP = for profit; na = not available; F&V: fruit and vegetables. Food: other food.

**Table 2:** Socio-demographic characteristics of food co-op members and non-members.

Characteristics	All		Members *N* = 51		Non-Members *N* = 254		*p*-Value *
	n	%	*n*	%	*n*	%	
**Total**	**305**	**96.2**	**51**	**16.7**	**254**	**83.3**	
**Women**	297	97.4	51	100	246	96.9	0.438
**Age group**	305						0.132
Less than 30 years	16	5.2	0	0	16	6.3	
30–44 years	232	76.1	43	84.3	189	74.4	
45 + years	57	18.7	8	15.7	49	19.3	
**SES based on postcode**	304						0.146
1st decile (most disadvantaged)	2	0.7	0	0	2	0.8	
2nd decile	5	1.6	1	2.0	4	1.6	
3rd decile	3	1.0	0	0	3	1.2	
4th decile	6	2.0	1	2.0	5	2.0	
5th decile	7	2.3	2	3.9	5	2.0	
6th decile	34	11.2	6	11.8	28	11.1	
7th decile	20	6.6	5	9.8	15	5.9	
8th decile	16	5.3	6	11.8	10	4.0	
9th decile	136	44.7	25	49.0	111	43.9	
10th decile (most advantaged)	75	24.7	5	9.8	70	27.7	
**Language spoken at home**	305						0.069
English	233	76.4	44	86.3	189	74.4	
Other	72	23.6	7	13.7	65	25.6	
**Aboriginal/Torres Strait Islander**	305						0.493
Yes	6	2.0	0	0	6	2.4	
No	295	96.7	50	98.0	245	96.5	
Unknown	4	1.3	1	2.0	3	1.2	
**Marital status**	305						0.125
Married/Partner	285	93.4	48	94.1	237	93.3	
Divorced	6	2.0	1	2.0	5	2.0	
Widow/er	1	0.3	1	2.0	0	0	
Single	13	4.3	1	2.0	12	4.7	
**Number of children**	303						0.041
0	6	2.0	0	0	6	2.4	
1	101	33.3	12	23.5	89	35.3	
2	143	47.2	25	49.0	118	46.8	
3	39	12.9	11	21.6	28	11.1	
4	10	3.3	3	5.9	7	2.8	
5	3	1.0	0	0	3	1.2	
6	1	0.3	0	0	1	0.4	
**Employment status**	305						0.028
Full time	108	35.4	11	21.6	97	38.2	
Part time	103	33.8	25	49.0	78	30.7	
Casual	33	10.8	2	3.9	31	12.2	
Unemployed	9	3.0	3	5.9	6	2.4	
Retired	1	0.3	0	0	1	0.4	
Home duties	51	16.7	10	19.6	41	16.1	
**Highest education level**	305						0.374
Year 10 or below	9	3.0	0	0	9	3.5	
Year 12	14	4.6	1	2.0	13	5.1	
TAFE or Diploma	56	18.4	11	21.6	45	17.7	
Higher education	226	74.1	39	76.5	187	73.6	

* *p*-value for difference between members and non-members of co-ops. Note: *p*-value was performed using independent *t*-test for continuous variable (number of children) or Pearson’s Chi-Square test for categorical variables (all other variables).

**Table 3:** Participant fruit and vegetable intake and related perceptions by fruit and vegetable co-op membership.

Intake and Perceptions	All		Members *N* = 51		Non-Members *N* = 254		*p*-Value
	n	%	n	%	n	%	
**Serves of vegetables per day**	305						
0 (I don’t eat vegetables)	1	0.3	0	0	1	0.4	0.007
0.5 (Less than 1 serve)	10	3.3	1	2.0	9	3.5	
1 serve	48	15.7	4	7.8	44	17.3	
2 serves	84	27.5	11	21.6	73	28.7	
3 serves	89	29.2	17	33.3	72	28.3	
4 serves	49	16.1	11	21.6	38	15.0	
5 serves	12	3.9	4	7.8	8	3.1	
6 or more serves	12	3.9	3	5.9	9	3.5	
**Perception of current vegetable consumption**	305						
Too little	169	55.4	25	49.0	144	56.7	0.436
About right	128	42.0	25	49.0	103	40.6	
Too much	3	1.0	0	0	3	1.2	
Don’t know	5	1.6	1	2.0	4	1.6	
**Serves of fruit per day**	305						
0 (I don’t eat vegetables)	7	2.3	0	0	7	2.8	0.801
0.5 (Less than 1 serve)	50	16.4	12	23.5	38	15.0	
1 serve	88	28.9	10	19.6	78	30.7	
2 serves	104	34.1	19	37.3	85	33.5	
3 serves	41	13.4	7	13.7	34	13.4	
4 serves	11	3.6	3	5.9	8	3.1	
5 serves	2	0.7	0	0	2	0.8	
6 or more serves	2	0.7	0	0	2	0.8	
**Perception of current fruit consumption**	305						
Too little	134	43.9	19	37.3	115	45.3	0.798
About right	151	49.5	30	58.8	121	47.6	
Too much	12	3.9	1	2.0	11	4.3	
Don’t know	8	2.6	1	2.0	7	2.8	
**Meet Australian guidelines for 5 serves vegetable daily**	305						
No	281	92.1	44	86.3	237	93.3	0.089
Yes	24	7.9	7	13.7	17	6.7	
**Meet Australian guidelines for 2 serves fruit daily**	305						
No	145	47.5	22	43.1	123	48.4	0.490
Yes	160	52.5	29	56.9	131	51.6	
**Meet Australian guidelines for fruit and vegetables daily**	305						
No	294	96.4	47	92.2	247	97.2	0.075
Yes	11	3.6	4	7.8	7	2.8	

**Table 4:** Comparison of odds of meeting the Australian guidelines for fruit and vegetable intake for food co-op members compared to non-members.

Outcomes		Model 1 OR (95% CI)	*p*-Value	Model 2 OR (95% CI)	*p*-Value	Model 3 OR (95% CI)	*p*-Value	Model 4 OR (95% CI)	*p*-Value	Model 5 OR (95% CI)	*p*-Value
Meets vegetable guideline	Members	2.22 (0.87–5.66)	0.096	2.48 (0.94–6.53)	0.067	2.11 (0.79–5.61)	0.137	2.22 (0.82–5.98)	0.115	2.11 (0.79–5.61)	0.137
	Non-members	1.0		1.0		1.0		1.0		1.0	
Meets fruit guideline	Members	1.24 (0.68–2.27)	0.491	1.27 (0.69–2.34)	0.451	1.21 (0.65–2.25)	0.546	1.22 (0.65–2.30)	0.534	1.22 (0.65–2.30)	0.534
	Non-members	1.0		1.0		1.0		1.0		1.0	
Meets both fruit and vegetable guideline	Members	3.00 (0.85–10.67)	0.089	4.77 (1.15–19.86)	0.032	4.28 (1.02–18.01)	0.047	4.74 (1.07–20.99)	0.041	4.77 (1.15–19.86)	0.032
	Non-members	1.0		1.0		1.0		1.0		1.0	

Model 1: unadjusted; Model 2: adjusted for age and education; Model 3: Model 2 + adjusted for language; Model 4: Model 3 + adjusted for marital status and number of children; Model 5: the model of best fit for each outcome.

## Supplementary Materials

The following are available online at https://www.mdpi.com/1660-4601/17/11/4154/s1, Demographic questions; Table S2. Fruit and vegetable related questions; Table S3. Food co-operative questions.
