# Supplementary material for: Food Co-Operatives: A Potential Community-Based Strategy to Improve Fruit and Vegetable Intake in Australia
Source: Int J Environ Res Public Health. 2020 Jun 10;17(11):4154. doi: 10.3390/ijerph17114154 (PMC7312496; doi:10.3390/ijerph17114154)
Supplement: Supplementary file 1 [file ijerph-17-04154-s001.pdf]

Table S1. Demographic Questions.

Category	Question	Responses
Age	What is your age?	15–19<br>20–24<br>25–29<br>30–34<br>35–39<br>40–44<br>45–49<br>50–54<br>55+
Gender	What is your gender?	Male<br>Female<br>Prefer not to say
SES	What is the postcode where you usually live? e.g. 2006 (Enter postcode in the box below)	Free numeral box
Ethnicity	Do you speak a language other than English at home?	No, English only.<br>Yes, (please specify)<br>Free text box
Aboriginal or Torres Strait Islander	Are you of Aboriginal or Torres Strait Islander origin?	Yes<br>No<br>Don't know
Education	What is the highest level of education that you have completed?	I didn't go to school<br>Year 10 (4th form) or below<br>Year 12 (6th form)<br>Certificate I, II, III, IV, Diploma, Advanced Diploma or Associate Degree<br>Graduate Diploma or Graduate Certificate<br>Bachelor or Postgraduate Degree (Masters or PhD)<br>Unsure (please describe what education you have completed)<br>Free text box
Marital status	What is your current relationship status?	Married/partner<br>Divorced<br>Widow/er<br>Single
Offspring 1	Do you have any children?	Yes<br>No
Offspring 2	If yes, how many children do you have?	Free numeral box
Employment 1	What is your current employment status?	Full time<br>Part time<br>Casual<br>Unemployed<br>Retired<br>Home duties

Table S2. Fruit and Vegetable Related Questions.

Category	Question	Responses
Vegetable serves 1	How many serves of vegetables do you usually eat each day?<br><br>(The picture shows a serve of vegetables. A serve is also = ½ cup cooked vegetables, or 1 cup of salad vegetables. Include fresh, dried, frozen and tinned vegetables)	I don't eat vegetables<br>Less than 1 serve<br>1 serve<br>2 serves<br>3 serves<br>4 serves<br>5 serves<br>6 or more serves
Vegetable serves 2	Would you say the amount of vegetables you currently eat is …	Too little<br>About right<br>Too much<br>Don't know
Fruit serves 1	How many serves of fruit do you usually eat each day?<br><br>(The picture shows a serve of fruit. A serve is also = 1 cup of diced pieces. Include fresh, dried, frozen and tinned fruit)	I don't eat fruit<br>Less than 1 serve<br>1 serve<br>2 serves<br>3 serves<br>4 serves<br>5 serves<br>6 or more serves
Fruit serves 2	Would you say the amount of fruit you currently eat is …	Too little<br>About right<br>Too much<br>Don't know

Table S3. Food Co-operative Questions.

Category	Question	Responses
Food co-operative 1	Are you a member of a food co-operative?<br><br>(Community based groups of people who come together voluntarily to purchase fresh fruit and vegetables or other foods in bulk)	Yes<br>No
Food co-operative 2	If yes, how many people are in your food co-operative?	Free numeral box<br>Don't know option
Food co-operative 3	If yes, what do you normally purchase?	Check boxes all that apply<br>Fruit<br>Vegetables<br>Other food items
Food co-operative 4	If yes, how often do you pick up a food box?	Greater than once per week<br>Once per week<br>Once per fortnight<br>Once per month<br>Less than once per month
Food co-operative 5	If yes, what dollar amount do you spend per food box? (AU$)	Free numeral box
